# Efficacy and safety of acupuncture on oligomenorrhea due to polycystic ovary syndrome: An international multicenter, pilot randomized controlled trial

**DOI:** 10.1097/MD.0000000000028674

**Published:** 2022-02-18

**Authors:** Kyoung Sun Park, Weijuan Gang, Pyung-Wha Kim, Changsop Yang, Purumea Jun, So-Young Jung, Ojin Kwon, Jin Moo Lee, Hye Jeong Lee, Su Jeong Lee, Xianghong Jing, Ning Zhang, Jing Hu, Jiping Zhao, Ran Pang, Chunlan Jin, Jun-Hwan Lee

**Affiliations:** a Jaseng Hospital of Korean Medicine, Seoul, Republic of Korea; b Institute of Acupuncture and Moxibustion, China Academy of Chinese Medical Sciences, Beijing, People's Republic of China; c Clinical Medicine Division, Korea Institute of Oriental Medicine, Daejeon, Republic of Korea; d University of Science & Technology, Campus of Korea Institute of Oriental Medicine, Korean Convergence Medicine Major, Daejeon, Republic of Korea; e Department of Obstetrics & Gynecology, College of Korean Medicine, Kyung Hee University, Seoul, Republic of Korea; f Dongzhimen Hospital Affiliated to Beijing University of Chinese Medicine, Beijing, People's Republic of China; g Guang An Men Hospital, China Academy of Chinese Medical Sciences, Beijing, People's Republic of China.; h Aerospace Center Hospital, Beijing, People's Republic of China.

**Keywords:** acupuncture, oligomenorrhea, polycystic ovary syndrome, protocol, randomized controlled trial

## Abstract

**Background:**

Polycystic ovary syndrome (PCOS) is one of the most common disorders of reproductive endocrinology affecting women of reproductive age. Our study aims to explore the feasibility of a full-scale trial to evaluate the efficacy and safety of acupuncture for PCOS.

**Methods:**

This study is a two-armed, parallel, multi-country, multi-center, pilot randomized controlled trial (RCT) for PCOS with oligomenorrhea. We will recruit 60 women aged 20 to 40 years with oligomenorrhea due to PCOS. The participants will be randomly assigned to acupuncture and control groups. The acupuncture group will undergo a total of 40 sessions for 16 weeks with usual care. The control group will be managed with usual care (regular meals, sufficient sleep, and appropriate exercise) only. The primary clinical outcome is mean change in menstrual frequency from baseline to 16 weeks and 32 weeks (follow-up) after the start of the trial. The secondary outcomes are menstrual period, levels of estradiol, luteinizing hormone (LH), follicle-stimulating hormone (FSH), and total testosterone, LH/FSH ratio, antral follicle count and ovarian volume, body mass index, waist hip ratio, acne severity, and health-related quality of life questionnaire scores at 16 and 32 weeks after the start of the trial.

**Discussion:**

This is the first protocol for multi-country, multi-center RCTs for PCOS in Korea and China. The control group in this study will be subjected to usual care (regular meals, enough sleep, and appropriate exercise). The results of this study will provide evidence for future clinical decisions and guidelines.

This trial has been registered at ClinicalTrials.gov (Identifier: NCT04509817).

## 1 Introduction

Polycystic ovary syndrome (PCOS) is one of the most common disorders of reproductive endocrinology affecting women of reproductive age. PCOS is diagnosed by the presence of any 2 of the following features: oligo- and/or anovulation, clinical and/or biochemical hyperandrogenism, and ultrasonography findings of polycystic ovaries, with the exclusion of other etiologies.^[1]^ Generally, the prevalence of PCOS is considered between 6% and 10%.^[2]^ PCOS is estimated to account for more than 75% of anovulatory infertility cases.^[3]^

The current first-line pharmacological therapy for PCOS patients is the oral administration of selective estrogen receptor modulators such as clomiphene.^[4]^ However, it is ineffective in 40% of the patients diagnosed with PCOS and is associated with significant side-effects like headaches, bloating, mood swings, and breast tenderness.^[5]^ The use of metformin as an insulin-sensitizing agent is also increasing, but the details of the mechanism underlying its efficacy remain elusive.^[6]^ A recently updated review suggested that the efficacy of metformin alone or combined with clomiphene was inconclusive, and the evidence quality was low.^[7]^ Moreover, combined oral contraceptive pills (OCPs) such as Diane-35 (ethinylestradiol and cyproterone acetate) are used by women with PCOS for controlling menstrual abnormalities, hirsutism, and acne.^[8]^ However, the use of OCPs is inappropriate for women who wish to conceive.^[9]^ OCPs may increase the risk of cardiovascular complications or aggravate obesity, thereby exacerbating the associated signs and symptoms of PCOS.^[10]^

Previous studies have found that acupuncture might be effective in improving menstrual (which means increase and regularization of menstrual period) frequency, ovulation rate, and serum hormone levels.^[^11^–^13^]^ A systematic review demonstrated that acupuncture appears to significantly improve the recovery of menstrual cycles and decrease the body mass index (BMI) and luteinizing hormone (LH) levels in women with PCOS.^[14]^ Meanwhile, another systematic review showed significant pooled benefits of acupuncture treatment, used as an adjunct to medication, on LH level, LH/follicular stimulating hormone (FSH) ratio, testosterone level, fasting insulin level, and pregnancy rates, but the level of evidence was low.^[15]^ The objective of this pilot trial is to explore the feasibility of a full-scale trial evaluating the efficacy and safety of acupuncture in improving menstrual frequency and alleviating other symptoms in patients with PCOS. The hypothesis is that acupuncture treatment is more effective than usual care. We will examine this hypothesis through a multi-country, multicenter, pilot randomized controlled trial (RCT).

## 2 Methods

### 2.1 Study design and setting

**Figure 1:**
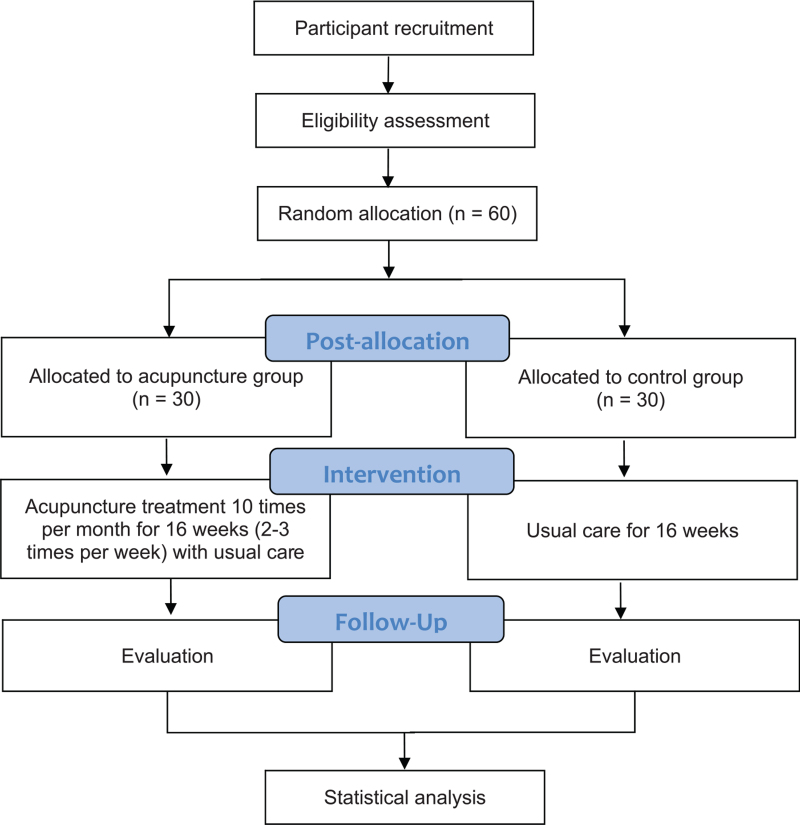
Flowchart of the trial.

This multi-country, multi-center, assessor-blinded RCT will be performed at the following three hospitals: Acupuncture and Moxibustion Hospital of China Academy of Chinese Medical Sciences (CACMS; Beijing, China), Dongzhimen Hospital (Beijing, China), and Kyung Hee University Hospital at Gangdong (Seoul, Republic of Korea). The study period will be 32 weeks, including 16 weeks of intervention and 16 weeks of follow-up. The study plan is summarized in Figure 1. This study protocol followed the Standard Protocol Items: Recommendations for Interventional Trials guidelines (SPIRIT).

### 2.2 Recruitment

Advertisements will be placed in the local newspaper, on the online advertising platforms and on the homepages/social media platforms of the hospitals to recruit participants. Additionally, posters and brochures will be put up in the 3 hospitals.

### 2.3 Eligibility criteria

#### 2.3.1 Inclusion criteria

The inclusion criteria are as follows:

1. oligomenorrhea (menstrual cycle >35 days or <8 cycles per year) with at least one of the following 2 criteria:
  A. Hyperandrogenism (clinical, biochemical, or both);
  B. Polycystic ovarian morphologic features (≥12 antral follicles of 2–9 mm diameter in either ovary; an ovarian volume **≥**10 mL in either ovary; or both);
2. Age 20 to 40 years;
3. Voluntary agreement to participation in the trial and provide informed consent prior to treatment.

#### 2.3.2 Exclusion criteria

We will exclude women who meet any of the following conditions:

1. Pregnancy, labor, or breastfeeding within the past 3 months;
2. Intake of oral contraceptives or ovulation-inducing agents within the past 3 months;
3. Severe oligomenorrhea with a menstrual cycle lasting >3 months;
4. Menstruation lasting >8 days;
5. Premature ovarian failure;
6. Resistant thyroid disease, Cushing's disease, congenital adrenal hyperplasia, or hyperprolactinemia;
7. Ovarian tumors or adrenal tumors that cause hyperandrogenemia;
8. Hemorrhagic disease;
9. Severe cardiac, pulmonary, hepatic, or renal diseases, central nervous system disorders, or intake of psychoactive drugs;
10. Acupuncture treatment within the past 1 month;
11. Participation in other clinical trials within the past 3 months;
12. Any other conditions considered inappropriate for inclusion in the trial by the investigators.

### 2.4 Interventions

This study protocol follows the Standards for Reporting Interventions in Clinical Trials of Acupuncture (STRICTA) recommendations. The participants will be randomly assigned to an acupuncture group or a control (usual care) group in a ratio of 1:1. They will receive interventions and undergo assessments at scheduled visits. Acupuncture will be performed according to a fixed protocol by Korean and Chinese medicine practitioners who have official licenses and at least 2 years of clinical experience. Patients in the acupuncture group will undergo acupuncture therapy 10 times in 4 weeks for 16 weeks, resulting in a total of 40 sessions. Disposable, sterilized, stainless steel single-use needles (0.25 × 40 mm and 0.30 × 50 mm; Dongbang Medical Co. Ltd, Republic of Korea and Qingdao Dongfang Medical Equipment Co., Ltd., China, respectively) will be inserted at a depth of 10 to 50 mm at acupuncture points located in the head, abdomen, lower back, and upper and lower limbs. In total, 18 needles will be used for each participant in a set. The acupoints include GV20 (Baihui), CV4 (Guanyuan), and bilateral BL32 (Ciliao), ST25 (Tianshu), ST29 (Guilai), EX-CA1 (Zigong), LI4 (Hegu), LR3 (Taichong), SP6 (Sanyinjiao), and SP9 (Yinlingquan) and are based on the traditional medicine theory and consensus among experts about acupuncture and PCOS.^[16]^ The location of the acupuncture points is based on the “Nomenclature and location of acupuncture points”^[17]^ and the consensus of experts. All needles will be stimulated manually by rotation until a needle sensation (“de-qi”) is evoked. The patient will be in the prone position first, and the needle will be inserted at the BL32 acupoint at an angle of 30 to 45° in an inferomedial direction and a depth of 30 to 50 mm. Once the patient feels the “de-qi,” the needle will be immediately removed. The patient will then be shifted to the supine position, and GV20, CV4, ST25, ST29, EX-CA1, LI4, LR3, SP6, and SP9 will be needled to a depth of 10 to 40 mm. After the “de-qi” sensation is felt by the patient, the needles will be kept in place for 20 minutes to maintain stimulation. Furthermore, patients will be advised to manage themselves with usual care, similar to that used by the control group.^[18]^

The participants in the control (usual care) group will be managed with the usual care regimen only. They will receive health advice and lifestyle management brochures regarding diet along with regular meals, sufficient sleep, and appropriate exercise once a month for 16 weeks.

The participants are not permitted concomitant medication like OCPs or ovulation induction that may affect menstrual cycles. And, acupuncture, moxibustion, cupping, and other traditional Chinese/Korean medicine treatments are neither allowed if they are judged to affect the outcome measures.

Adherence to acupuncture will be checked every 4 weeks during the treatment period, and participants who have an adherence rate of less than 70% will be dropped out.

### 2.5 Outcomes

The time points for measuring the outcomes are presented in detail in Table 1. This study is preliminary, but will be allowed for an extensive evaluation of clinical outcome measures as follows:

**Table 1:**
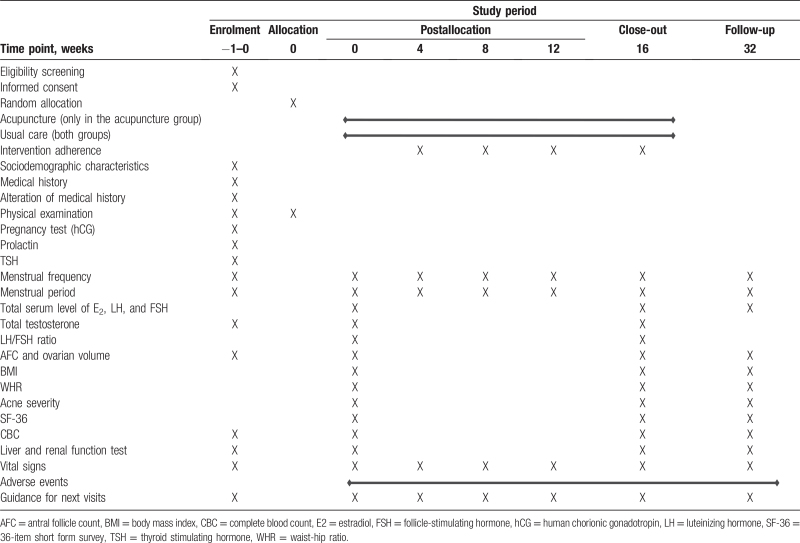
Schedule of enrolment, interventions, and assessments.

#### 2.5.1 Primary outcome

The primary outcome is the mean change in menstrual frequency (menstrual cycle/month) from baseline to 16 weeks and 32 weeks (follow-up) after the start of the trial. The menstrual frequency will be assessed to evaluate the improvement in ovulatory functions and restoration of menstruation of PCOS patients.^[19]^ The average menstrual frequency for the last year will be recorded as the baseline evaluation. The average menstrual frequency for 16 weeks during the treatment period will be recorded at a close-out evaluation, and that for another 16 weeks during the follow-up period will be recorded at a follow-up evaluation.

#### 2.5.2 Secondary outcomes

The secondary outcomes include mean changes in menstrual period, levels of estradiol, LH, FSH, and total testosterone, LH/FSH ratio, antral follicle count in the ovary, ovarian volume, BMI, waist-hip ratio (WHR), acne severity, and 36-item short-form health survey (SF-36) scores from baseline to 16 weeks and 32 weeks after the start of the trial.

The menstrual period is defined as the number of days from the first day of the menstrual cycle to the first day of the next menstrual cycle. It is assessed in order to evaluate the restored regular menstrual period.^[16]^ The levels of serum estradiol, serum LH, serum FSH, and total testosterone, and LH/FSH ratio will be estimated to evaluate the participant's sex hormone changes after interventions.^[^2^,^^20^^]^ The morphologic features of polycystic ovaries will be assessed by antral follicle count and ovarian volume via ultrasound imaging. A transvaginal transducer with frequencies of ≥8 MHz detects follicles with diameters in the range of 2 to 9 mm in the ovary and can be used to estimate ovarian volume.^[21]^ Follicular excess and ovarian enlargement are considered accurate markers of polycystic ovarian morphology.^[2]^ The participants’ obesity level will be assessed using BMI and WHR. BMI is defined as the body weight (kg) divided by the square of the height (m^2^). WHR is calculated using the circumference of the waist and hip.^[^22^,^^23^^]^ The acne severity will be assessed using the Pillsbury acne grading system, with grades ranging from 0 to 4. A high-scoring grade indicates more severe acne, that is, grade 0 is considered normal and grade 4 is considered to be severe.^[24]^ We will also assess the quality of life as reflected by an SF-36 score for the participants. The SF-36 questionnaire has been proved to be valid and reliable in each country. It will be used to assess the health-related quality of life of women with PCOS.^[^25^,^^26^^]^ The SF-36 questionnaire is a 36-item, patient-reported survey of patient health consisting of eight subscales, with a total score ranging from 0 to 100. The higher the score, the lesser the disability, that is, a score of 0 is equivalent to maximum disability and that of 100 is equivalent to no disability.^[27]^

### 2.6 Safety and adverse events

Complete blood count and liver and renal functions will be examined to determine the safety of the acupuncture technique at screening and at 16 and 32 weeks after the start of the trial. Laboratory data will be recorded in a case report form (CRF) by the investigators. All expected or unexpected adverse events in both groups will be assessed at every visit. The participants will be asked to record any adverse events in their CRF throughout the study. All adverse events will be described in the CRF. Participants who suffer from the adverse effects of acupuncture will be treated or compensated according to insurance regulations.

### 2.7 Participant timeline

The schedule for enrollment, interventions, assessment, and visits of the participants are shown in Table 1.

### 2.8 Sample size

We calculated the sample size based on a previous study that used the same variables as ours as the primary outcome.^[12]^ For the current study, the mean difference and standard deviation are assumed as 0.14 and 0.17, respectively, between the acupuncture and control groups. In other words, the effect size for menstrual frequency is assumed to be approximately 0.8. In our study, participants will be randomized into 2 groups in a 1:1 ratio after considering 5% of the significance level (α) and 80% of the power (1−β). Therefore, the present study will include 50 participants in total, divided into 2 groups of 25. Considering a dropout rate of 15%, at least 60 participants are required.

### 2.9 Randomization and allocation concealment

In this trial, participants will be randomly assigned to either the acupuncture group or control group in a 1:1 ratio using a table of random numbers. An independent, blinded statistician will generate random numbers using the block randomization method of SAS version 9.4 (SAS Institute. Inc., Cary, NC) with a block size of 4. The investigator will be subsequently notified of the number assigned to each participant, and the participants will be given a random number on their second visit. The allocation table of participants will be kept by an independent statistician until the end of the study. The clinical research coordinator (CRC) will enroll the participants, generate the allocation sequence, and assign participants to the intervention groups.

### 2.10 Blinding

This is an open-label trial; therefore, neither the patients nor the practitioners will be blinded to the entire process. However, other researchers, including outcome assessors and statisticians, will be blinded to eliminate potential bias. Assessors, who are not involved in randomization or acupuncture performance, will ask simple and necessary questions to participnats for outcome assessments so that they cannot assume the groups of each participant.

### 2.11 Data collection methods and management

All research assistants, including the CRCs at the 3 centers, will be trained using a standard operating procedure to promote data quality. The laboratory data will be analyzed in the medical laboratories of South Korea and China that meet the international standard ISO 15189^[^28^,^^29^^]^ to reduce errors between each center.

Data collection procedures will be conducted in compliance with the approved protocol and the standard operating procedure of the trial to ensure good data quality. All data, including the CRF and worksheet, will be kept in a cabinet with locking devices. At the end of the trial, all data written in the CRF will be coded in separate computer files. Data entry will be performed using a double-input method, and the matching work will be conducted after reviewing inconsistent data. When the data are matched, a data clarification form will be completed and validated, and the resolution will be reflected in the data. Computer files in computer storage devices will be kept confidential under password protection during and after the trial.

### 2.12 Data and safety monitoring

A quality monitoring committee will be established independent of the researchers and sponsors. All data will be verified to ensure that they are recorded and reported accurately and completely and are consistent with the original data. The principal investigator will be responsible for monitoring the entire process. The entire study process will be under strict supervision. All adverse events and other unintended effects of acupuncture or trial conduct will be collected by the CRC. The principal investigators at each trial site, who will be notified by the CRC, will assess whether the adverse events are relevant to acupuncture and whether the trial should be amended or ended.

### 2.13 Statistical methods

All statistical analyses will be performed using SAS version 9.4 (SAS Institute. Inc., Cary, NC) with a two-tailed test. The level of significance will be set at 5%. Missing data will be substituted using multiple imputations.

The analysis set will consists of a full analysis set (FAS), per-protocol (PP) set, and safety set. The main analyses will be conducted using FAS. A FAS is described as set that is as complete and close as possible to the intention-to-treat. The efficacy test will be assessed by FAS analysis and PP analysis. The safety test will be assessed by intent-to-treat analysis, as well as by PP analysis.

Baseline characteristics of the groups with continuous data will be compared using an independent *t*-test or a Wilcoxon rank-sum test. Baseline characteristics with categorical data will be compared using a Chi-Squared test or Fisher exact test.

The primary outcome measure in this study will be the mean change in menstrual frequency from baseline to 16 and 32 weeks after the start of the trial. To analyze menstrual frequency as the primary outcome, we will perform an analysis of covariance considering the baseline menstrual frequency as the covariate and treatment group as the fixed factor. Additionally, intra-group menstrual frequency changes from baseline to post-treatment will be analyzed using a paired *t*-test or Wilcoxon signed-rank test.

The methods used to analyze the secondary outcome measures will be the same as those used for the primary outcome analyses. If the data type is categorical, it will be analyzed using a Chi-Squared test or Fisher exact test.

## 3 Discussion

Acupuncture is widely practiced and has now been utilized in the Western countries as a treatment or adjunct treatment of an increasing number of conditions. It is a relatively safe treatment with few side effects.^[30]^ Acupuncture activates and modulates the nervous pathways at peripheral (local), segmental (in the spinal cord), and supraspinal levels within the central nervous system. The hypothetical mechanism of the effects of acupuncture in PCOS is that stimulation of acupuncture needles in the skeletal muscles excites ergoreceptors that activate afferent sensory nerve fibers. These signals are transmitted to the spinal cord, where they may modulate the sympathetic output to the target organs in the same area of innervation through spinal reflexes. Signals also reach the central nervous system via supraspinal pathways, and here they can exert central effects. Hypothalamic β-endorphin has been implicated in the effects of acupuncture. It modulates the autonomic system but may also alter the release of gonadotropin-releasing hormone and corticotropin-releasing hormone. These hormones exert an effect on reproductive function (via LH and FSH), adrenal function (adrenocorticotropic hormone), and pancreatic function (circulating β-endorphins).^[31]^

In a recent Cochrane review, true acupuncture did not show clinically relevant differences in the live birth rate, multiple pregnancy rate, ovulation rate, clinical pregnancy rate, or miscarriage rate when compared to sham acupuncture.^[19]^ Most previous RCTs examined the efficacy of acupuncture compared to sham acupuncture, but the appropriate control group in acupuncture trials is debatable. This is because non-penetrating or superficial acupuncture is not completely inert.^[32]^ According to a review of acupuncture trials, most studies found no statistically significant difference in the outcomes, and sham acupuncture could be as efficacious as true acupuncture, especially when superficial needling is applied to nonpoints. There is nothing sham about inserting needles into a patient, and it appears that researchers conducting acupuncture trials may be ignorant about how acupuncture works, or they may have employed a control treatment that cannot be scientifically distinguished from the intervention.^[33]^

Sham acupuncture is useful in RCTs in which subjective outcomes are affected by the success of blinding. In our trial, objective outcomes and menstrual frequency, which might be relatively less affected by whether blinding is performed or not, will be used. In the aforementioned Cochrane review, low-frequency electroacupuncture was more effective in restoring the regular menstrual period than physical exercise or no intervention was in PCOS patients, but the quality of the evidence was very low.^[19]^ Therefore, an RCT to examine the efficacy of acupuncture compared to that of physical exercise or no intervention is needed. In our study, we will use participants undergoing usual care (regular meals, sufficient sleep, and appropriate exercise) as the control group. Moreover, this is the first multi-country, multi-center RCT conducted for PCOS in Korea and China. The results of this study will provide evidence for guiding future clinical decisions and guideline development, as well as for a full-scale clinical trial.

## Author contributions

**Conceptualization:** Kyoung Sun Park, Weijuan Gang.

**Funding acquisition:** Chunlan Jin, Jun-Hwan Lee.

**Investigation:** Jin Moo Lee, Hye Jeong Lee, Su Jeong Lee, Xianghong Jing, Ning Zhang, Jing Hu, Jiping Zhao, Chunlan Jin.

**Methodology:** Pyung-Wha Kim, Changsop Yang, Purumea Jun, So-Young Jung, Ojin Kwon, Jin Moo Lee, Hye Jeong Lee, Su Jeong Lee, Xianghong Jing, Ning Zhang, Jing Hu, Ran Pang, Jiping Zhao.

**Software:** Ojin Kwon.

**Supervision:** Chunlan Jin, Jun-Hwan Lee.

**Writing – original draft:** Kyoung Sun Park, Weijuan Gang.

**Writing – review & editing:** Pyung-Wha Kim, Hye Jeong Lee, Jiping Zhao, Chunlan Jin, Jun-Hwan Lee.
